# Should prophylaxis of venous thromboembolism in Asian patients undergoing knee and hip arthroplasty and hip fracture surgery be an issue?

**DOI:** 10.1186/s43019-021-00105-3

**Published:** 2021-07-29

**Authors:** Aree Tanavalee

**Affiliations:** Chulalongkorn University Faculty of Medicine, 1873 Rama IV Road, Pathumwan, Bangkok, 10330 Thailand

Postoperative venous thromboembolism (VTE) is a significant cause of morbidity and mortality in patients undergoing knee and hip arthroplasty and hip fracture surgery, while VTE is considered potentially preventable with several modalities of prophylactic management [1].

The VTE prevention guidelines by the American Academy of Orthopedic Surgeons (AAOS) or the American College of Chest Physicians (AACP) have been implemented in most countries in Asia [2, 3]. However, there are some concerning issues and complications related to VTE prophylaxis in major joint replacement and hip fracture surgeries according to these guidelines, due to differences in the healthcare systems and various cultural aspects [4–6].

Among orthopedic surgeons who practice in the Asia-Pacific (AP) region, some alternative options for VTE prevention in hip and knee arthroplasty and hip fracture surgery are believed to be necessary [7–10]. These Asian-specific guideline/consensus statements are expected to provide better patient outcomes and compliance. Therefore, in a 1-year period, The Thai Hip and Knee Society (THKS) has initiated an AP consensus agreement on VTE prophylaxis in knee and hip arthroplasty and hip fracture surgery in Asian patients. Ninety-three orthopedic experts from the AP region volunteered to join the consensus using an overall five-round modified Delphi method. According to the results of these AP consensus statements, one should be aware that some agreed methods of VTE prophylaxis are different from those published in international guidelines regarding diagnosis and risk factors and methods of prophylaxis in details.

We hope that this AP VTE consensus will provide orthopedic surgeons who practice in the AP region appropriate options for VTE diagnosis and prevention methods that benefit their patients, with fewer complications.

## References

[1] Edelsberg J, Ollendorf D, Oster G (2001). Venous thromboembolism following major orthopedic surgery: review of epidemiology and economics. Am J Health Syst Pharm.

[2] Barrack RL (2012). Current guidelines for total joint VTE prophylaxis: dawn of a new day. J Bone Joint Surg (Br).

[3] Flierl MA, Messina MJ, Mitchell JJ, Hogan C, D’Ambrosia R (2015). Venous thromboembolism prophylaxis after total joint arthroplasty. Orthopedics.

[4] Yassin M, Mitchell C, Diab M, Senior C (2014). The necessity of pharmacological prophylaxis against venous thromboembolism in major joint arthroplasty. Int Orthop.

[5] Bala A, Murasko MJ, Burk DR, Huddleston JI, Goodman SB, Maloney WJ, Amanatullah DF (2020). Venous thromboprophylaxis after total hip arthroplasty: aspirin, warfarin, enoxaparin, or factor Xa inhibitors?. Hip Int.

[6] Matharu GS, Garriga C, Whitehouse MR, Rangan A, Judge A (2020). Is aspirin as effective as the newer direct oral anticoagulants for venous thromboembolism prophylaxis after total hip and knee arthroplasty? An analysis from the National Joint Registry for England, Wales, Northern Ireland, and the Isle of Man. J Arthroplast.

[7] Cha SI, Lee SY, Kim CH, Park JY, Jung TH, Yi JH, Lee J, Huh S, Lee HJ, Kim SY (2010). Venous thromboembolism in Korean patients undergoing major orthopedic surgery: a prospective observational study using computed tomographic (CT) pulmonary angiography and indirect CT venography. J Korean Med Sci.

[8] Won MH, Lee GW, Lee TJ, Moon KH (2011). Prevalence and risk factors of thromboembolism after joint arthroplasty without chemical thromboprophylaxis in an Asian population. J Arthroplast.

[9] Bin Abd Razak HR, Soon AT, Dhanaraj ID, Tan AH (2012). Incidence of clinically significant venous thromboembolic events in Asian patients undergoing total knee arthroplasty without anticoagulation. J Arthroplast.

[10] Cho KY, Kim KI, Khurana S, Bae DK, Jin W (2013). Is routine chemoprophylaxis necessary for prevention of venous thromboembolism following knee arthroplasty in a low incidence population?. Arch Orthop Trauma Surg.

